# An Adaptive Attention-Driven Quadruplet Deep Hashing Method for Retrieving Histopathological Images

**DOI:** 10.3390/jimaging12070321

**Published:** 2026-07-15

**Authors:** Seyed Mohammad Alizadeh, Henning Müller, Mohammad Sadegh Helfroush

**Affiliations:** 1 Department of Electrical Engineering, Shiraz University of Technology, Shiraz 71557-13876, Iran; ms_helfroush@sutech.ac.ir; 2 Informatics Institute, University of Applied Sciences Western Switzerland (HES-SO), Rue du Technopole 3, 3960 Sierre, Switzerland; 3 Medical Faculty, University of Geneva, 1211 Geneva, Switzerland

**Keywords:** attention module, deep hashing, histopathological image retrieval, quadruplet models, medical images

## Abstract

Retrieving histopathological images can assist in the recognition and treatment planning of several diseases. Nevertheless, high-dimensional features can make this process complex and inefficient. These challenges can be addressed by encoding the feature domain into binary codes of different lengths utilizing deep hashing approaches. Still, the vanishing gradient challenge remains a concern in these approaches. According to several studies, quadruplet deep hashing models have exhibited promising performance in retrieving images from multi-category datasets. Furthermore, adding an attention module to a convolutional neural network architecture can increase the efficiency of feature extraction. Thus, we introduce an adaptive quadruplet deep hashing model to retrieve histopathological images. Four designed deep hashing models with matching structures and parameters are utilized to produce hash codes. The resulting codes are trained according to a novel adaptive quadruplet loss function. The adaptive structure is capable of improving retrieval performance. The presented approach also suggests a novel hash layer for the vanishing gradient issue. In addition, a simple yet effective attention module is implemented to enhance feature extraction performance. Our model is evaluated on three publicly available histopathology datasets: Kather, Kimia Path960, and Kimia Path24C. The results indicate that the suggested approach achieves the highest mean average precision (MAP) of approximately 0.9940, 0.9983, and 0.9968 for the respective datasets. Based on experiments performed on the datasets, our model surpasses current hashing techniques.

## 1. Introduction

Retrieving histopathological images can aid in recognizing a variety of diseases, including different types of cancers [1]. After a query image is input, a retrieval process organizes the dataset images by similarity using high-dimensional features, which can be time-consuming and complicated [2]. To address these challenges, hashing techniques map feature domains into binary representations of various lengths [3]. Deep hashing approaches usually outperform classical ones by utilizing pairwise, triplet, and quadruplet structures [4]. However, developing and learning binary codes can be a challenge because of the vanishing gradient issue [3]. According to recent research, quadruplet deep hashing models outperform pairwise or triplet algorithms for image retrieval in datasets with multiple categories [5].

The effectiveness of convolutional neural networks (CNNs) for classifying and retrieving histopathology images has been demonstrated in many studies [6,7]. As CNNs become more sophisticated, there is no statistically significant increase in their effectiveness, resulting in the development of attention mechanisms [8]. In a way similar to how human eyes operate, CNNs can focus on key features and content using the attention mechanism [9]. Integrating the attention mechanism into a deep hashing scheme improves the efficiency of an image retrieval procedure [10].

While various models for retrieving histopathology images have been proposed recently, they may not be sufficiently accurate for datasets with many categories and diverse images [11]. Furthermore, the design of a quadruplet deep hashing model incorporating attention mechanisms seems promising to improve histopathology image retrieval [12,13]. In this paper, we present a deep hashing approach utilizing an adaptive quadruplet structure to retrieve histopathology images, called histopathology attention quadruplet deep hashing (HAQDH). This study makes the following main contributions:

- A novel adaptive quadruplet deep hashing attention-based model to retrieve histopathological images is introduced for the first time.
- An efficient hash layer is proposed for tackling the vanishing gradient issue.
- We present an adaptive quadruplet loss function that boosts retrieval performance and also contributes to generating high-accuracy binary codes.
- A simple yet effective attention module is provided to focus on more details in histopathology images, improving the feature extraction process.
- Experimental results show that HAQDH outperforms the most advanced hashing-based techniques on three public histopathology datasets.

The paper proposes a novel deep hashing approach based on attention that is less complex, more accurate, and faster compared to other approaches. Our proposed hash layer produces binary codes with high accuracy, addressing the vanishing gradient issue. The presented quadruplet model can also help identify the most similar images to a query by accurately comparing binary codes. Furthermore, our simple attention module improves the performance of the feature extraction stage. The above contributions have all been achieved by introducing new parameters and factors rather than merely combining existing approaches, leading to high-performance retrieval of histopathological images.

The remainder of this paper is organized as follows. Various attention mechanisms, hashing techniques, and histopathology image retrieval methodologies are discussed in Section 2. A detailed explanation of the presented approach is provided in Section 3. In Section 4, we summarize and analyze our findings. Section 5 concludes with a brief overview of the paper, its limitations, and future study recommendations.

## 2. Related Work

### 2.1. Hashing Strategies

Hashing strategies, which can be classified into traditional and deep learning-based models, facilitate the retrieval of images with minimal storage requirements [1]. Locality-sensitive hashing (LSH) [14], iterative quantization (ITQ) [15], and supervised discrete hashing (SDH) [16] are the most widely used traditional hashing techniques. LSH represents the feature space in a data-independent form by employing stochastic hash functions. ITQ eliminates the quantization separation of the feature vector and the generated hash codes. SDH utilizes an approach based on regression for developing binary values.

Recently, several advanced deep hashing techniques have been introduced for image retrieval. Deep pairwise-supervised hashing (DPSH) [17] utilizes two identical CNNs for generating binary codes learned by a novel loss function, providing encouraging results. Deep supervised hashing (DSH) employs a type of contrastive loss function to train generated hash values [18]. While DPSH and DSH are fundamental deep hashing models, their accuracy in image retrieval may be limited [19]. Deep triplet supervised hashing (DTSH) [20] and deep triplet quantization (DTQ) [21] employ a triplet structure to train binary codes. HashNet presents a new hash layer using a scaled tanh to tackle the vanishing gradient problem [3]. Improved deep hashing network (IDHN) is a technique suggested by Zhang et al. [4] to facilitate image retrieval in multi-category datasets by comparing pair binary values based on their quantitative similarity. In [5], a deep architecture, named quadruplet deep hashing (QDH), is proposed that learns hash values via a quadruplet scheme. The model may not be sufficiently accurate in retrieving all types of images. By leveraging the attention algorithm integrated into a triplet hashing strategy, attention-based triplet hashing (ATH) improves the efficiency of retrieving medical images [2]. Nevertheless, ATH does not utilize various datasets, which can be a limitation. Opponent class adaptive margin (OCAM) develops a triplet hashing algorithm incorporating an adjustable margin to retrieve medical images [22]. Cheng et al. [23] propose a deep hashing network that employs a nonlinear attention mechanism. While the model demonstrates acceptable performance in retrieving images on certain datasets, further testing on additional datasets is necessary for a more comprehensive evaluation of its effectiveness. In [19], an autoencoder-based deep hashing model is proposed for general image retrieval. However, the method may lack sufficient accuracy.

### 2.2. Attention Mechanisms

CNNs can be tuned to focus on crucial information (channel attention) and location (spatial attention) based on attention methods [8,24]. In fact, attention modules can help select the best region of an image, leading to the selection of the best features [25]. Additionally, some features may be unnecessary for our purpose and can be disregarded by the attention mechanisms [26]. A number of attention modules have been designed in recent years to improve the feature extraction performance of CNNs [27]. The convolutional block attention module (CBAM) was proposed in [8], utilizing channel and spatial attention strategies in a sequential order. In [25], the efficient channel attention (ECA) module was presented for increasing CNN efficiency while maintaining low sophistication. The Style-based Recalibration Module (SRM) improved CNN representing capability by adding the relative significance of distinct styles into feature representations [28]. A hybrid attention module was developed by Li et al. [9], following a framework similar to ECA and CBAM in the channel attention and spatial phases, respectively.

### 2.3. Histopathological Image Retrieval

An unsupervised strategy was suggested by Ma et al. [29] for retrieving histopathology images to diagnose breast cancer. The approach employed LSH to optimize the retrieval process. To retrieve and classify histology images, Shi et al. [1] presented a pairwise deep hashing method with an original objective function. Gu et al. [11] introduced a new deep hashing strategy to retrieve histopathological images with an emphasis on breast cancer recognition. Sukhia et al. [30] suggest a retrieval approach employing local features and vector of locally aggregated descriptors (VLADs) coding. Another hashing-based CBHIR model was proposed in [31]. The authors applied LSH and ITQ, which did not yield encouraging results in retrieving histopathological images. Yang et al. [12] developed an attention-based deep metric learning technique for retrieving histopathology images. The employed attention mechanism could help highlight relevant regions, leading to encouraging results. A Siamese deep hashing algorithm was designed in [32]. The authors presented a unique loss function to further optimize the retrieval effectiveness. In [33], a fusion model is presented to retrieve histopathology images. Most introduced models do not achieve high accuracy in retrieving histopathology images. Therefore, presenting a model that is both highly accurate and less complex would be useful.

## 3. Methods

**Figure 1:**
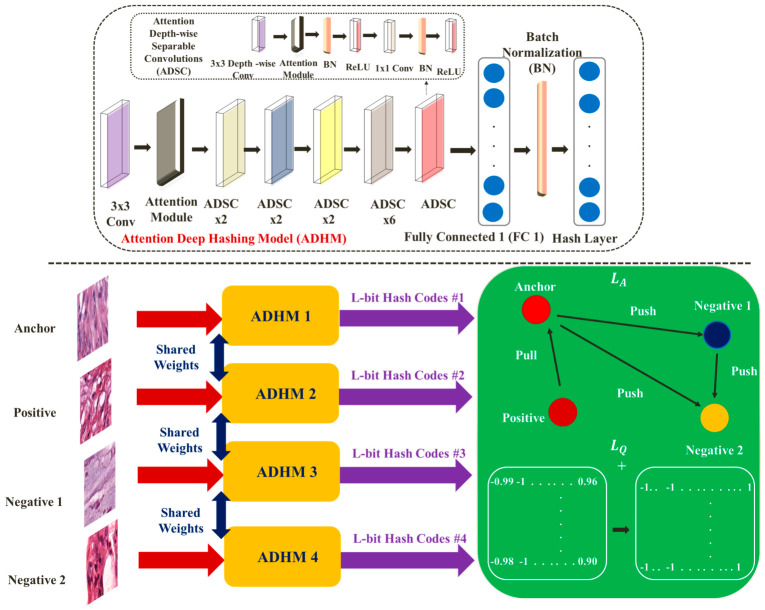
A visual description of the presented model. The method contains four designed deep hashing models with matching structures and parameters, named ADHM 1–4. The feature extraction process is improved by the attention module developed.

Based on Figure 1, HAQDH comprises four corresponding deep hashing models, named ADHM 1–4, with the following inputs: anchor, positive, negative1, and negative2. The class labels of anchors and positives are the same, while those of negatives are different. Additionally, negatives1 and negatives2 do not belong to the same category. The four images above are chosen at random based on similar studies such as [2,5]. First, we represent each image *x_i_* using a single M-bit hash code $\;h_{i}\in \;{\left\{-1,1\right\}}^{M}$. The produced codes must then be learned through the suggested loss function. Binary codes are compared using a novel quadruplet loss function (green part of Figure 1). Furthermore, the accuracy of generated hash codes is controlled in this part. This section explains the framework and creation of deep hashing designs, the training step, and the retrieval procedure.

### 3.1. Deep Hashing Models Framework

Since MobileNet [34] performs well in histopathology image analysis and attention mechanism study, it is selected as the primary network for feature extraction [6,8]. We utilized a pre-trained classical model of MobileNet, which was fine-tuned on our datasets based on previous studies [35,36]. The feature extraction process is improved with the help of an attention module. After explaining the presented attention mechanism, we describe the overall design of deep hashing. The four deep hashing models share the same weights and structure.

#### 3.1.1. Presented Attention Module

**Figure 2:**
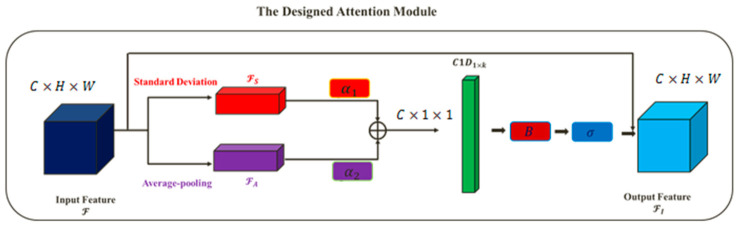
The structure of our designed attention module. The standard deviation and average-pooling functions are first used to collect feature information globally. The outputs are then combined utilizing two trainable parameters. Finally, a 1-D convolutional layer is applied with kernel k.

Taking inspiration from [28], we present an updated version of the ECA module. As indicated in Figure 2, the standard deviation and average-pooling functions are first utilized to collect feature details globally, leading to $F_{S}\in \;R^{C\times 1\times 1},$ and $F_{A}\in \;R^{C\times 1\times 1}$. The outputs are then added together employing the trainable parameters $\alpha_{1}$ and $\alpha_{2}$. Finally, a 1-D convolutional layer is applied with kernel *k*, calculated as follows [25]:

(1) $$k={\left|\frac{{log}_{2}(number\;of\;channels)}{2}+\;\frac{1}{2}\right|}_{odd}$$

where ${\left|z\right|}_{odd}$ denotes the nearest odd number to *z*. Accordingly, the improved features ($F_{I}$) is calculated as follows:

(2) $$F_{I}=F⊗(\sigma (B({C1D}_{1\times k}(\alpha_{1}⨂F_{S}⨁\;\alpha_{2}⨂F_{A}))))\;\;\;\;\;\;\;s.t.\;\;\alpha_{1}+\alpha_{2}=1\;$$

where $F\in \;R^{C\times H\times W},$ σ, B, C1D, ⨂ and ⨁ denote the input features, sigmoid function, batch normalization, 1-D convolutional layer, element-wise multiplication, and element-wise addition, respectively. It should be mentioned that C, H, and W show channel, height, and width dimensions, respectively.

#### 3.1.2. Deep Hashing Design

In order to design our deep hashing model, we replace the softmax layer with a fully connected one comprising M nodes in the attention-based CNN model (Figure 1). Additionally, a parametric Softsign activation function is presented for producing hash codes to avoid the vanishing gradient challenge. As a result, an image can be encoded in M-bit binary format according to the following:

(3) $$h=Softsign\;\alpha (WF+V)=\frac{\alpha \left(WF+V\right)}{\left(1+\alpha \left|WF+V\right|\right)}$$

where W *∈* $R^{M\times 512}$, F *∈* $R^{512\times 1}$*,* and V *∈* $R^{M\times 1}$ refer to the weight matrix, generated features, and bias vector, respectively. Furthermore, the value of α is determined to be 10 based on the results of the experiment.

### 3.2. The Proposed Loss Function

Let $h_{A}$,$\;h_{P}$, $h_{N_{1}}$ and $h_{N_{2}}$ be the binary values produced for anchor, positive, negative1 and negative2 samples, respectively. For each quadruplet pattern i, inspired by [13], we propose th ion:

(4) $$L_{A}=\sum_{i}\begin{array}{c} (\max\left({∥h_{A_{i}}-\;h_{P_{i}}∥}_{2}^{2}\;-\;{∥h_{A_{i}}-\;h_{N_{i_{1}}}∥}_{2}^{2}\;\;+\lambda_{AD1}\;,\;0\right)\\ +\max\left({∥h_{A_{i}}-\;h_{P_{i}}∥}_{2}^{2}\;-\;{∥h_{A_{i}}-\;h_{N_{i_{2}}}∥}_{2}^{2}\;+\lambda_{AD2}\;,\;0\right)\\ +\max\left({∥h_{A_{i}}-\;h_{P_{i}}∥}_{2}^{2}\;-\;{∥h_{N_{i_{1}}}-\;h_{N_{i_{2}}}∥}_{2}^{2}\;\;+\lambda_{AD3}\;,\;0\right)) \end{array}$$

where ${∥\;.∥}_{2}$ denotes the Euclidean distance. Furthermore, $\lambda_{AD}$ is calculated for two binary codes *I_i_* and *I_j_* as follows:

(5) $$\lambda_{AD}=\beta \left(e-e^{S\left(I_{i},\;I_{j}\right)}\right)\;\;\;\;s.t.\;\;\;\;0<\beta <1$$

where $S\left(I_{i},\;I_{j}\right)$ denotes the Cosine similarity between *I_i_* and *I_j_*.

Our hash layer may not provide pure binary codes, so we include a quantization loss factor defined as follows:

(6) $$L_{Q}=\begin{array}{c} \sum_{i}\;({∥{sign\;(h}_{A_{i}})-\;h_{A_{i}}∥}_{2}^{2}\;+\;{∥{sign\;(h}_{P_{i}})-\;h_{P_{i}}∥}_{2}^{2}\\ +{∥{sign\;(h}_{N_{i_{1}}})-\;h_{N_{i_{1}}}∥}_{2}^{2}+\;{∥{sign\;(h}_{N_{i_{2}}})-\;h_{N_{i_{2}}}∥}_{2}^{2}) \end{array}$$

Accordingly, the total loss function is:

(7) $${\;\;\;L}_{T}={\;L}_{A}+{\gamma L}_{Q}$$

where γ is employed to regulate *L_Q_*.

The learning stage attempts to minimize our loss function by employing the back-propagation methodology. Hence, the derivative of our loss function can be calculated accordingly:

(8) $$\frac{\partial L_{T}}{\partial h_{A_{i}}}=\frac{\partial L_{A_{1}}}{\partial h_{A_{i}}}+\frac{\partial L_{A_{2}}}{\partial h_{A_{i}}\;}+\frac{\partial L_{A_{3}}}{\partial h_{A_{i}}}+\gamma \frac{\partial L_{Q}}{\partial h_{A_{i}}}$$

where

(9) $$\frac{\partial L_{A_{1}}}{\partial h_{A_{i}}}=\left\{\begin{array}{c} \sum_{i}2*\left(h_{A_{i}}-h_{P_{i}}\right)-2*\left(h_{A_{i}}-h_{{N_{i}}_{1}}\right),\;\;{∥h_{A_{i}}-h_{P_{i}}∥}_{2}^{2}>{∥h_{A_{i}}-h_{{N_{i}}_{1}}∥}_{2}^{2}-\lambda_{AD1}\\ \;\;\;\;\;\;\;\;\;\;\;\;\;0\;\;\;\;\;\;\;\;\;\;\;\;\;\;\;\;\;\;\;\;\;\;\;\;\;\;\;\;\;\;\;\;\;,\;\;otherwise \end{array}\right.$$

(10) $$\frac{\partial L_{A_{2}}}{\partial h_{A_{i}}}=\left\{\begin{array}{c} \sum_{i}2*\left(h_{A_{i}}-h_{P_{i}}\right)-2*\left(h_{A_{i}}-h_{{N_{i}}_{2}}\right),\;\;{∥h_{A_{i}}-h_{P_{i}}∥}_{2}^{2}>{∥h_{A_{i}}-h_{{N_{i}}_{2}}∥}_{2}^{2}-\lambda_{AD2}\\ \;\;\;\;\;\;\;\;\;\;\;\;\;0\;\;\;\;\;\;\;\;\;\;\;\;\;\;\;\;\;\;\;\;\;\;\;\;\;\;\;\;\;\;\;\;\;,\;\;otherwise \end{array}\right.$$

(11) $$\frac{\partial L_{A_{3}}}{\partial h_{A_{i}}}=\left\{\begin{array}{c} \sum_{i}2*\left(h_{A_{i}}-h_{P_{i}}\right),\;\;{∥h_{A_{i}}-h_{P_{i}}∥}_{2}^{2}>{∥h_{{N_{i}}_{1}}-h_{{N_{i}}_{2}}∥}_{2}^{2}-\lambda_{AD3}\\ \;\;\;\;\;\;\;\;\;\;\;\;\;0\;\;\;\;\;\;\;\;\;\;\;\;\;\;\;\;\;\;\;\;\;\;\;\;\;\;\;\;\;\;\;\;\;,\;\;otherwise \end{array}\right.$$

(12) $$\frac{\partial L_{Q}}{\partial h_{A_{i}}}=\sum_{i,j}-2\left({sign\;(h}_{A_{i}})-h_{A_{i}}\;\right)$$

Following that, the chain rule is employed to determine the gradients of *L_T_* concerning the relevant variables:

(13) $$\frac{\partial L_{T}}{\partial W}=\frac{\partial L_{T}}{\partial h_{A_{i}}}\frac{\partial h_{A_{i}}}{\partial W}=(\frac{\partial L_{T}}{\partial h_{A_{i}}})\frac{F}{{\left(\left|WF+V\right|+1\right)}^{2}}$$

(14) $$\frac{\partial L_{T}}{\partial F}=\frac{\partial L_{T}}{\partial h_{A_{i}}}\frac{\partial h_{A_{i}}}{\partial F}=(\frac{\partial L_{T}}{\partial h_{A_{i}}})\frac{W}{{\left(\left|WF+V\right|+1\right)}^{2}}$$

(15) $$\frac{\partial L_{T}}{\partial V}=\frac{\partial L_{T}}{\partial h_{A_{i}}}\frac{\partial h_{A_{i}}}{\partial V}=(\frac{\partial L_{T}}{\partial h_{A_{i}}})\frac{1}{{\left(\left|WF+V\right|+1\right)}^{2}}$$

These stages can also be carried out for other produced binary values. By using the standard back-propagation algorithm, all parameters can be optimized. Back-propagation is a technique utilized to compute gradients for training a neural network and update its parameters. Back-propagation calculates the gradient of a loss function relative to the network parameters efficiently. It achieves this by propagating derivatives backward, layer by layer, preventing unnecessary chain-rule computations [37]. It is important to note that the back-propagation algorithm is a widely used technique in deep learning applications. This method is not original to us; we simply employ it to update parameters. Using this technique, Equations (8)–(15) are applied from the outer layer to the inner layer to minimize the loss function. Algorithm 1 describes the general HAQDH learning process.
**Algorithm 1:** The HAQDH training method**Input:** Histopathological images $X={\left\{x_{i}\right\}}_{i=1}^{M}$ **Output:** The parameters W, F, and V **Initialization:** Set up W and V **Repeat**
   1Create quadruplet patterns;   2**Find** F using the presented attention-based approach;   3Generate binary codes for samples of a quadruplet pattern using (3);   4Calculate derivatives according to (8)–(15);   5Adapt the parameters W, F **and** V employing the back-propagation training method; **Based on** a particular number of repetitions

### 3.3. Phase of Retrieval

This phase compares a query instance with the whole training list to identify corresponding samples. A binary code can be calculated directly for a sample *x_j_* by the sign function as follows:

(16) $$h_{x_{j}}=sign\;(WF_{x_{j}}+V)$$

## 4. Experimental Results

### 4.1. Datasets

Our work utilizes three publicly available histopathological datasets, Kather [38], Kimia Path960 [39], and Kimia Path24C [40]. Recent studies have demonstrated the significant potential of these datasets for content-based image retrieval [12,30,31,32,36]. It should be noted that all images are normalized before being fed into our model.

**Kather**: It contains 5000 histopathological images of 150 × 150 pixels used for colorectal cancer screening, organized into eight categories with the same sample count, consisting of tumor epithelium, simple stroma, complex stroma, immune cells, adipose tissue, normal mucosal, debris, and background (no tissue).

**Kimia Path960**: Over 400 whole slide images of connective tissue, epithelial, and muscle can be found in this dataset. In each image, 48 areas of interest are picked and downsampled, resulting in 960 patches with a size of 308 × 168.

Inspired by [30,31,36], in both datasets, the training and test samples are randomly distributed with a 70% to 30% ratio for each class. The average findings from five experiments are presented.

**Kimia Path24C**: This dataset is an updated representation of Kimia Path24, which was first introduced in [41]. It includes 22,591 training and 1325 test patches, each 1000 × 1000 in size, taken from 24 whole slide images representing various areas of the body.

### 4.2. Details of Implementation

In this study, the tests are performed on a computer with a 2.20 GHz CPU, 32 GB of RAM, and a Tesla T4 GPU utilizing the Python 3.11 deep learning package Keras. The adaptive moment estimation (Adam) optimizer is used [42] based on a learning rate of 0.001 and a batch size of 64.

According to similar research, such as [1,4,32], we compare our model to the state-of-the-art hashing methods, including LSH, ITQ, HashNet, ATH, DSH, DPSH, DTQ, OCAM, and IDHN. For the datasets utilized, however, some parameters may need to be adapted to improve the results. This paper adheres to the same method as earlier similar works to provide a fair comparison [4]. Additionally, all aspects related to selecting datasets and parameters have been kept consistent for a fair comparison.

### 4.3. Metrics

Per previous research works [4], this study employs the respective assessment metrics:

**Mean Average Precision (MAP)**: For a query, this statistic represents the mean of the average precision (AP), computed as follows:

(17) $$AP=\frac{1}{N_{f}}\sum_{k}^{N_{g}}\frac{N_{k}}{k}\;\times \;S_{k}$$

$N_{g}$ denotes the database size, $N_{f}$ denotes the number of fitting images, and $N_{k}$ denotes the number of relevant samples appearing in the top k rankings. In addition, and $S_{k}\;$ is 1 if the returned sample corresponds to the query; otherwise, it is 0.

**Precision@r:** The precision of finding the top *r* images corresponding to the query sample can be defined in the form of:

(18) $$Precision@r=\frac{1}{r}\sum_{k=1}^{r}S_{k}$$

### 4.4. Results

**Figure 3:**
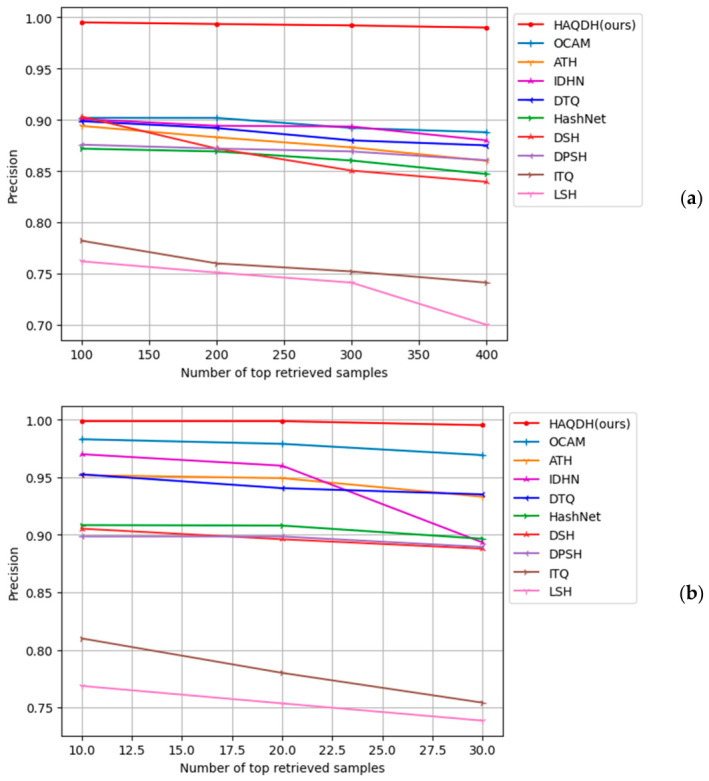

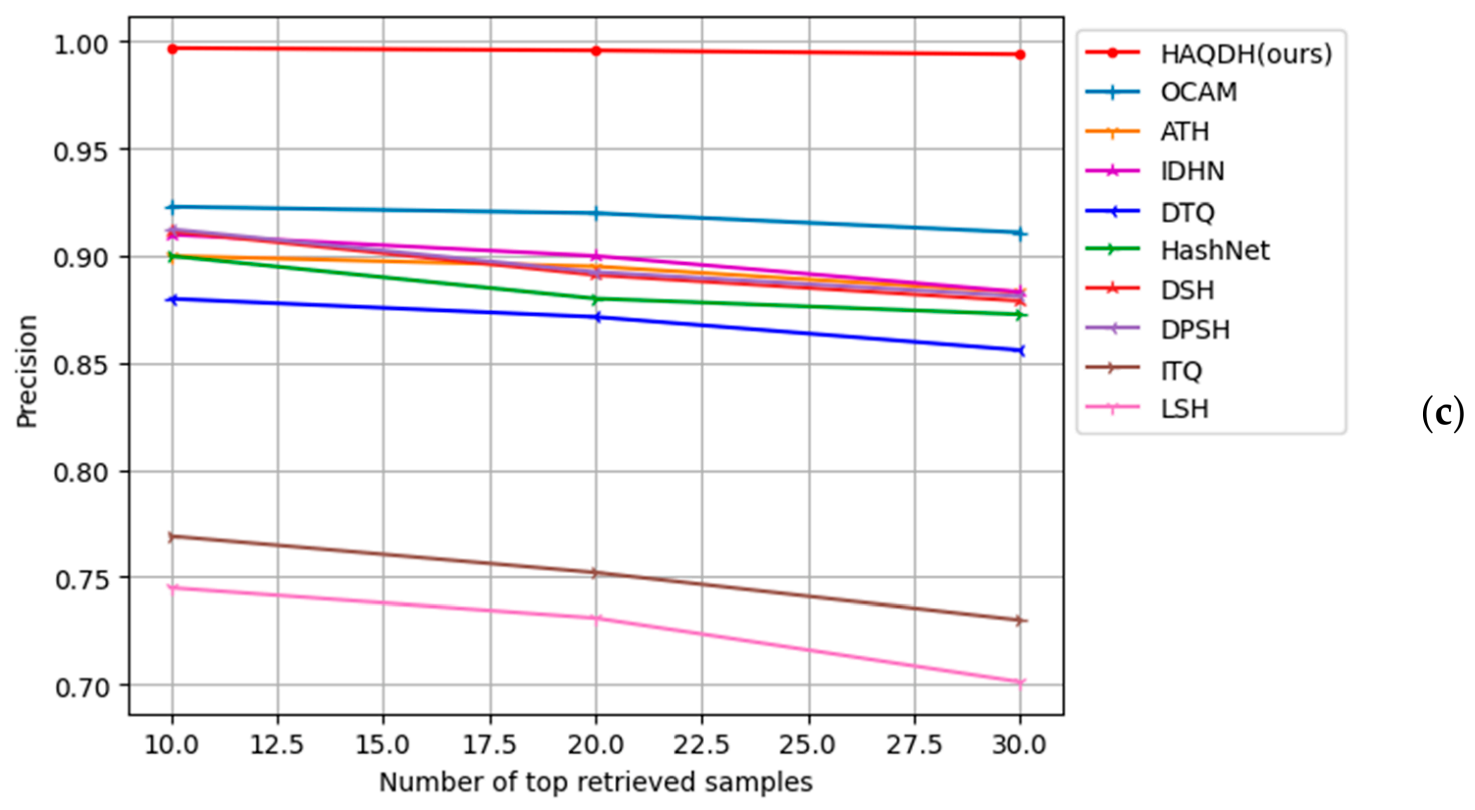
Precision curves for varying numbers of retrieved samples for the datasets employed ((**a**) Kather, (**b**) Kimia Path960, (**c**) Kimia Path24C).

As shown in Table 1, Table 2 and Table 3, HAQDH outperforms state-of-the-art hashing methods for several binary value lengths based on MAP for the three datasets. Compared to OCAM, the most efficient hashing algorithm, our model increases MAP by approximately 8–10%, 3–5%, and 5–8% on Kather, Kimia Path960, and Kimia Path24C, respectively. As an additional evaluation of the effectiveness of the methodologies, we present the precision curves for several numbers of retrieved samples. According to Figure 3, our proposed model is highly accurate for different numbers of retrieved histopathological images for the datasets used.

### 4.5. Ablation Study

**Figure 4:**
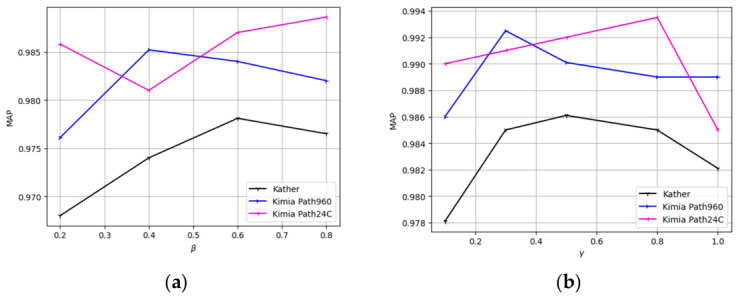
Examining the impact of the applied hyperparameters on retrieval performance (for simplicity, γ is considered 0 for all datasets in (**a**). β is considered 0.6, 0.4 and 0.8 for Kather, Kimia Path24C and Kimia Path960 in (**b**), respectively).

This paper proposes an adaptive quadruplet loss function to increase performance in the training and retrieval stages. The loss function contains two hyperparameters, β and γ. Figure 4a,b illustrates the β and γ effects estimated by MAP for the three datasets. It can be seen that the best MAP for Kather is achieved when β and γ are approximately equal to 0.6 and 0.5, respectively. The values for Kimia Path960, however, are around 0.4 and 0.3. Furthermore, these values are about 0.8 and 0.8 for Kimia Path24C. For the sake of brevity, we report the results for a 64-bit binary code.

The performance of the presented loss function is evaluated by replacing it with two loss functions proposed in [5,22]. These variants are referred to as HAQDH-V1 and HAQDH-V2, respectively. According to Table 4, our loss function performs better than its rivals. In addition, HAQDH-V3, HAQDH-V4 and HAQDH-V5 substitute our attention module with ECA, CBAM, and SRM, respectively. The findings of this study indicate that the designed attention module can facilitate the feature extraction process for our CNN model. To evaluate the proposed hash layer, we replace it with those presented in [3,43] (HAQDH-V6 and HAQDH-V7). The results demonstrate that our hash layer generates binary codes with high accuracy compared to others, leading to enhanced retrieval performance. To study the effect of the presented attention mechanism module, we replace our attention-based CNN model with MobileNet and VGG19 [44] architectures without any attention modules, dubbed HAQDH-V8 and HAQDH-V9. The results show that the presented attention module can improve MAP by approximately 3.5–6.5%. It should be mentioned that in Table 4, we aim to highlight the best performance of the sub-models regardless of the dataset type in Table 4. As a result, we only mention the best MAP for an overall comparison to avoid redundancy.

### 4.6. Discussion

In this paper, we present an attention-based adaptive quadruplet deep hashing approach for retrieving histopathological images. With an adaptive quadruplet structure, samples can be distinguished more easily and accurately, leading to improved retrieval results compared to triplet and pairwise deep hashing models, especially in datasets with different classes. Considering Table 1, Table 2 and Table 3, our approach outperforms other state-of-the-art hashing approaches for histopathology image retrieval. Compared to OCAM, the best-performing method among those evaluated, HAQDH achieves an enhancement of roughly 4–8% in MAP across various datasets. Additionally, the developed attention module can improve feature extraction, leading to enhanced accuracy in the retrieval process (see the HAQDH-V1, HAQDH-V2 and HAQDH results in Table 4). HAQDH-V6 and HAQDH-V7 utilize different hash layers, as proposed in [3,43], rather than our hash layer. Table 4 demonstrates that HAQDH with our hash layer improves MAP by nearly 3–5% compared to our model using alternative hash layers.

**Figure 5:**
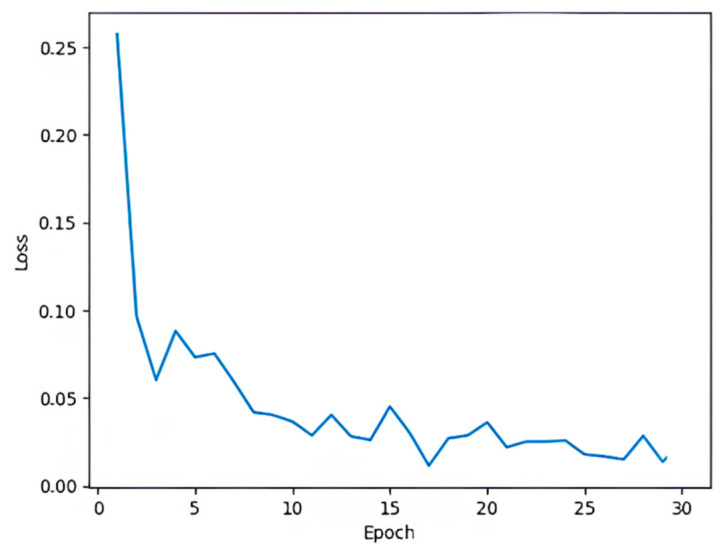
The convergence curves for the training process of our model on the Kather dataset.

In Table 5, the training and retrieval times of our model are compared with those of some deep hashing models to provide an overview of its performance in speed. As we do not consider image loading time in our calculations, the results are not very different for the three databases. As illustrated in Table 5, HAQDH demonstrates a rapid performance during the retrieval process. It is important to note that this comparison has been conducted under similar circumstances for a fair evaluation. As with previous studies [3,9,45], hyperparameters are typically set with an initial value and then adjusted based on a defined range during the learning process. Finally, the best value is determined by considering the results of each dataset. In the case of epochs, we used a range of 30 to 150 numbers, although 30 to 50 numbers are often adequate, depending on the datasets. A sample of the convergence curves for our model training on the Kather dataset is shown in Figure 5.

MAP is an evaluation metric used to evaluate a retrieval model and is not usually a metric for overfitting [2,19]. Nevertheless, similar to previous papers [2,46], we applied some techniques to avoid overfitting, including cross-validation, augmenting the datasets, using pre-trained models, employing dropout layers, applying regularization, and so on.

**Figure 6:**
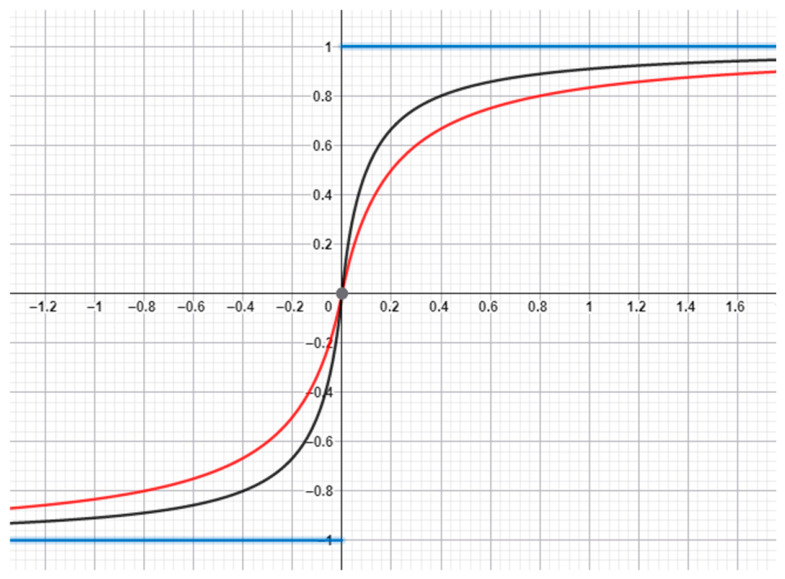
Comparing our activation function and the sign function (blue: the sign function, red: ours with α = 5, black: ours with α = 10).

Figure 6 provides a clear comparison of our activation function with the sign function for different α values. The plots show that our activation function can produce binary codes with high accuracy while effectively addressing the vanishing gradient issue. This is because the derivative of the Softsign function can be defined at zero, in contrast to the sign function. In this study, we focused on patch-based and small slide histopathology images while excluding whole slide images to reduce complexity, which may limit our work. Additionally, similar to [11], we concentrated on the computer vision aspect and utilized prepared datasets without direct collaboration with pathologists, which could be a concern. We plan to address these issues in future research.

In general, we present an attention-based quadruplet deep hashing model for retrieving histopathology images. This adaptive quadruplet structure is designed to be more efficient in recognizing both similar and dissimilar images. Our attention module is not only simple but also more effective compared to other existing attention modules. As indicated in Table 4, using our proposed attention module instead of popular alternatives can enhance MAP by almost 5%. The choice of parameters based on specific datasets and our objectives is a key factor in this improvement. Additionally, our hash layer effectively addresses the vanishing gradient problem, producing accurate binary codes. As shown in Table 4, this hash layer can improve MAP by approximately 4%.

### 4.7. Visual Retrieval Results

**Figure 7:**
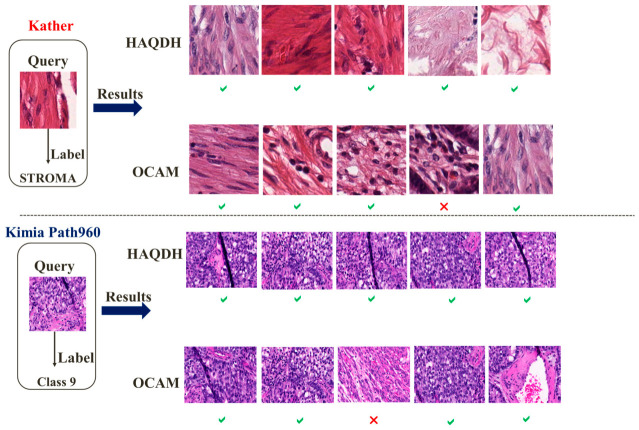
A visual comparison of retrieval results between our method and OCAM for the Kather and Kimia Path960 datasets.

Figure 7 displays five retrieved samples for our model and OCAM, as the best cutting-edge hashing model. According to the results, HAQDH outperforms OCAM in this experiment.

## 5. Conclusions

In this paper, we presented an attention-based adaptive quadruplet deep hashing method for retrieving histopathological images. Four deep hashing models with matching structures and parameters were utilized to create hash codes. The generated codes were trained with a newly developed adaptive quadruplet loss function. Furthermore, a simple and effective attention module was designed to be incorporated into our CNN model architecture to help increase the accuracy of the feature extraction procedure. The studies performed on three histopathology datasets have demonstrated that HAQDH outperformed state-of-the-art hashing approaches. The highest MAP of our model was nearly 0.997, 0.998, and 0.994 for the Kimia Path24C, Kimia Path960, and Kather datasets, respectively. The promising results indicate that our model can help diagnose diseases early, ultimately saving patients. Furthermore, this model can be developed for other modalities besides histopathology images, which can be useful in various applications.

In this paper, we presented a deep hashing model designed for the retrieval of histopathology images, yielding promising results that benefit both physicians and patients by reducing the need for biopsies and associated costs. However, it is important to note that this work was purely academic and focused on machine learning methods within a medical context. The primary goal was to introduce novel machine learning techniques.

In this study, we utilized well-known public histology datasets of appropriate size to avoid complexity. However, considering the use of larger databases in both size and quantity could be beneficial for future studies. Due to university restrictions and the unavailability of proper datasets and experts, this work focused on the pattern recognition and machine learning aspects. It followed similar studies that utilized prepared datasets without directly collaborating with clinicians. Nevertheless, this matter will be noted in our next research.

## Figures and Tables

**Table 1:** Kather MAP results of several hashing-based methods for different hash code lengths.

Approaches	32-Bits	64-Bits	128-Bits
HAQDH (ours)	0.9832 ± 0.012	0.9894 ± 0.007	0.9940 ± 0.004
OCAM	0.8883 ± 0.022	0.9078 ± 0.009	0.9149 ± 0.010
ATH	0.8859 ± 0.009	0.8947 ± 0.005	0.9057 ± 0.012
IDHN	0.8857 ± 0.018	0.8857 ± 0.018	0.8930 ± 0.024
DTQ	0.8705 ± 0.010	0.8790 ± 0.002	0.8868 ± 0.008
HashNet	0.8649 ± 0.021	0.8714 ± 0.016	0.8790 ± 0.014
DSH	0.8351 ± 0.026	0.8491 ± 0.021	0.8612 ± 0.017
DPSH	0.8602 ± 0.013	0.8693 ± 0.014	0.8713 ± 0.016
ITQ	0.7063 ± 0.058	0.7381 ± 0.041	0.7530 ± 0.038
LSH	0.7001 ± 0.068	0.7238 ± 0.039	0.7470 ± 0.045

**Table 2:** Kimia Path960 MAP results of several hashing-based methods for different hash code lengths.

Approaches	32-Bits	64-Bits	128-Bits
HAQDH (ours)	0.9849 ± 0.004	0.9908 ± 0.001	0.9983 ± 0.001
OCAM	0.9322 ± 0.019	0.9635 ± 0.016	0.9679 ± 0.015
ATH	0.9205 ± 0.007	0.9476 ± 0.022	0.9564 ± 0.018
IDHN	0.9446 ± 0.011	0.9590 ± 0.016	0.9666 ± 0.015
DTQ	0.9015 ± 0.005	0.9295 ± 0.010	0.9418 ± 0.012
HashNet	0.8709 ± 0.011	0.9070 ± 0.002	0.9136 ± 0.006
DSH	0.8649 ± 0.004	0.8885 ± 0.009	0.8963 ± 0.007
DPSH	0.8436 ± 0.008	0.8706 ± 0.012	0.8930 ± 0.010
ITQ	0.7250 ± 0.033	0.7581 ± 0.048	0.7728 ± 0.061
LSH	0.7192 ± 0.083	0.7401 ± 0.071	0.7589 ± 0.054

**Table 3:** Kimia Path24C MAP results of several hashing-based methods for different hash code lengths.

Approaches	32-Bits	64-Bits	128-Bits
HAQDH (ours)	0.9854	0.9935	0.9968
OCAM	0.9080	0.9303	0.9451
ATH	0.8503	0.8749	0.8952
IDHN	0.8867	0.9179	0.9344
DTQ	0.8383	0.8663	0.8902
HashNet	0.8412	0.8591	0.8731
DSH	0.8510	0.8636	0.8817
DPSH	0.8622	0.8748	0.8992
ITQ	0.6497	0.6873	0.7350
LSH	0.6255	0.6608	0.7168

**Table 4:** An overview of HAQDH and its variants.

Methods	Hash Layer	Attention Module	Loss Function	The Best MAP
HAQDH-V1	ours	ours	[22]	0.9589
HAQDH-V2	ours	ours	[5]	0.9698
HAQDH-V3	ours	ECA	ours	0.9543
HAQDH-V4	ours	CBAM	ours	0.9601
HAQDH-V5	ours	SRM	ours	0.9639
HAQDH-V6	[43]	ours	ours	0.9425
HAQDH-V7	[3]	ours	ours	0.9670
HAQDH-V8	ours	-	ours	0.9630
HAQDH-V9	ours	-	ours	0.9347
HAQDH	ours	ours	ours	0.9983

**Table 5:** A comparison of training and search times of our model and several deep hashing models for one iteration in seconds.

Approaches	Training	Search
HAQDH (ours)	183.15	0.85
ATH	294.35	2.25
HashNet	256.01	1.69
DSH	263.17	1.55

## Data Availability

The raw data supporting the conclusions of this article will be made available by the authors on request.
